# Lost Stones During Laparoscopic Cholecystectomy

**DOI:** 10.1155/1998/95874

**Published:** 1998-12

**Authors:** J. Diez, C. Arozamena, L. Gutierrez, J. Bracco, A. Mon, R. Sanchez Almeyra, M. Secchi

**Affiliations:** 1 Hospital de Clínicas Universidad de Buenos Aires Argentina; 2 Hospital Ramos Mejía Buenos Aires Argentina; 3 Clínica Pueyrredón Mar del Plata Argentina; 4 Hospital Británico Rosario Argentina; 5 Hospital Italiano Rosario Argentina; 6 Av. Quintana 70 Buenos Aires (1014) Argentina

## Abstract

*Background: * Gallbladder perforation, with loss of calculi in the abdomen is frequent during laparoscopic cholecystectomy. Recent publications report complications in port sites or in the abdominal cavity. A study of 3686 laparsocopic cholecystectomies performed by 6 surgeons was undertaken. In 627 patients, perforation of the gallbladder occurred and in 254 stones were spilled into the abdominal cavity. In 214 they were retrieved and in 40 left in the abdomen. Twelve patients developed complications. Percutaneous drainage was successful in 2 with serous collections. Two patients with abdominal abscesses were reoperated, stones retrieved and the abdomen drained.

One patient developed an intestinal obstruction due to a stone in the ileum. One patient who had a cholecystectomy in another hospital developed a paraumbilical tumor. At reoperation a stone was retrieved. In another six patients, stones were found in port sites. Stones lost into the abdomen should be removed because of their potential morbidity, especially if they are large or if infection is present in the gallbladder at the time of initial surgery. There is no indication for routine conversion to open surgery when stone spillage occurs, although patients should be informed to avoid legal consequence, and to hasten early diagnosis of later complications.


*HPB Surgery*, 1998, Vol. 11, pp. 105-109
Reprints available directly from the publisher
Photocopying permitted by license only

© 1998 OPA (Overseas Publishers Association) N.V.
Published by license under
the Harwood Academic Publishers imprint,
part of The Gordon and Breach Publishing Group.
Printed in Malaysia.

### Lost Stones During Laparoscopic Cholecystectomy

J. DIEZ [a,*], C. AROZAMENA [a], L. GUTIERREZ [b], J. BRACCO [c], A. MON [d], R. SANCHEZ ALMEYRA [d] and M. SECCHI [e]

[a] *Hospital de Clínicas, Universidad de Buenos Aires;* [b] *Hospital Ramos Mejía, Buenos Aires;* [c] *Clínica Pueyrredón, Mar del Plata;* [d] *Hospital Británico, Rosario;* [e] *Hospital Italiano, Rosario*

*(Received 6 June 1997; In final form 16 December 1997)*

***Background:*** **Gallbladder perforation, with loss of calculi in the abdomen is frequent during laparoscopic cholecystectomy. Recent publications report complications in port sites or in the abdominal cavity. A study of 3686 laparsocopic cholecystectomies performed by 6 surgeons was undertaken. In 627 patients, perforation of the gallbladder occurred and in 254 stones were spilled into the abdominal cavity. In 214 they were retrieved and in 40 left in the abdomen. Twelve patients developed complications. Percutaneous drainage was successful in 2 with serous collections. Two patients with abdominal abscesses were reoperated, stones retrieved and the abdomen drained.**

**One patient developed an intestinal obstruction due to a stone in the ileum. One patient who had a cholecystectomy in another hospital developed a paraumbilical tumor. At reoperation a stone was retrieved. In another six patients, stones were found in port sites. Stones lost into the abdomen should be removed because of their potential morbidity, especially if they are large or if infection is present in the gallbladder at the time of initial surgery. There is no indication for routine conversion to open surgery when stone spillage occurs, although patients should be informed to avoid legal consequence, and to hasten early diagnosis of later complications.**

*Keywords:* Laparoscopic cholecystectomy, lost stones

### INTRODUCTION

Gallbladder disruption with stones falling into the peritoneal cavity was rare during open cholecystectomy, but is frequent in laparoscopic cholecystectomy. Retrieval of stones is difficult during laparoscopic cholecystectomy.

Early reports on laparoscopic cholecystectomy stated that stones, left in the peritoneal cavity, had no deleterious effect [26, 31]. More recently as a consequence of spilled stones sepsis, adhesions and fistulae into abdominal organs or port tracts have been described [3, 6, 8, 14, 15]. In this paper we assessed the morbidity caused by stones abandones in the abdomen or port tracts.

### METHODS

In a retrospective study the records of 3686 patients submitted to laparoscopic cholecystectomy operated by 7 surgeons using similar

*Corresponding to: J. Diez, Av. Quintana 70, (1014) Buenos Aires, Argentina.

operative techniques between 1992 and 1995 were reviewed. Perforation of the gallbladder with spillage of stones and reports if they were retrieved or abandoned in the abdominal cavity were recorded in the operative records and in a special form recomended for laparoscopic biliary surgery by the Laparoscopic Commitee of the Argentine Asociation of Surgery. The gallbladder was extracted though the umbilical incision in all patients.

Ultrasonography, CT scans and fistulographies were the methods used in detection of stones.

### RESULTS

Perforation of the gallbladder occurred in 627 cases (17%) and in 254 (6.9%) stones spilled into the abdominal cavity. In 214 patients (5.8%) the stones were retrieved but in 40 (1%) the calculi were abandoned in the abdomen (Tab. I). Of these 12 developed complications. In 5 patients, the gallstones presented with intraabdominal problems. Four developed abdominal collections, two serous and two purulent. Six other patients in which no stones were abandoned had post-operative abdominal collections. In 3 bile was responsible and the other 3 were due to blood. All patients recovered with percutaneous drainage [3] or reoperation [3]. One patient had a small bowel obstruction due to a stone lost in the abdomen which entered into the ileum (Tab. II). In 7 patients the stones appeared in fistulous tracts in port sites: 6 at the umbilicus and 1 in a subxiphoid location. In one patient fistulography revealed communication with the transverse colon.

TABLE I Symptoms (*n* 12)

| | |
|---|---|
| Fever | 4 |
| Pain | 4 |
| Infected sinus tracts | 7 |
| Abdominal collections | 4 |
| Intestinal obstruction | 1 |
| Colonic fistula | 1 |

TABLE II Intraabdominal stones (*n* 5)

| | |
|---|---|
| Fever | 4 |
| Abdominal pain | 1 |
| Intestinal obstruction | 1 |
| Serous collections | 2 |
| Purulent collections | 2 |

### TREATMENT

In 2 patients the serous collections were evacuated percutaneously and the stone left in place. The patients remain asymptomatic for 1 and 2 years respectively.

In the 2 cases with purulent abscesses a laparotomy was performed, the abdominal cavity drained and the stones retrieved. An emergency operation was performed for the intestinal obstruction, and a large stone was found in the terminal ileum. The stone was removed and the perforation closed (Tab. III).

Of the 7 patients with stones in sinus or fistula tracts, in 4 the stones were retrieved at the skin and in 3 of them a surgical exploration was performed to extract the stones. All fistulous tracts and the colonic fistulae closed spontaneously (Tab. IV).

### DISCUSSION

Perforation of the gallbladder with bile and calculi falling into the abdomen is common in

TABLE III

| |
|---|
| Intraabdominal stones (*n* 5) |
| Treatment |
| Percutaneous drainage 2 |
| (stones left in place) |
| Surgical drainage of abdominal abscess |
| and recovery of stones 2 |
| Operation for ileal obstruction 1 |

TABLE IV Stones in port sites (*n* 7)

| | |
|---|---|
| Surgical exploration of the sinus tracts | 2 |
| Surgical exploration of subcutaneous tumor | 1 |
| Spontaneous cure after stones were expelled | 4 |

laparoscopic cholecystectomy [3, 8, 20, 23]. It occurs in 10% of cases [Strasberg, 22]. It was believed that stones left in the abdomen would not cause problems [19, 26, 31, 13]. Bile alone even infected, is not deleterious if aspirated, the cavity irrigated by saline solution, and antibiotic therapy instituted [8]. Recent reports by Catarci [3], Diez [8], Guy [10], Kent [13], Klaiber [14], Paul [17], Ponsky [19] and Targerona [23] report morbidity produced by abandoned stones and recommend that if possible all of them should be retrieved.

A variety of techniques and instruments are available to extract stones: ligature or clips on the gallbladder wall [20] aspiration of the small stones, [26] Dormia Baskets [14, 26] bags [13] finger gloves or condom [11, 13]. A 30 degree laparoscope is useful, it allows a better view of the reces between the bowel and the liver. Using those methods in 40 of our patients (1%) stones were lost in the abdomen and 12 of them caused complictions (25%).

Symptoms appeared from days to several months after the operation [3, 10]. Fever, pain and infection are common [1, 4].

The seriousness of the complications is different if the stones are in the abdominal cavity or in the port tracts.

Intraabdominal stones, produce serous or purulent collections and may cause generalized peritonitis, depending of the size of the stones [8, 21] and degree of bacterial contamination [33, 24]. Large or pigmented stones [3, 21] are more likely to produce these complications. Welch [27] in an experimental study demonstrated that some stones can shrink and even be reabsorbed. This might explain why many patients with lost stones remain asymptomatic.

Serous collections can be cured by percutaneous drainage, leaving the stones in the abdomen as occurred in our 2 cases. If the collection is purulent or generalized peritonitis is present, the abdomen should be explored, drained and stones removed either by laparoscopy or open laparotomy. Reoperation was successful in 2 of our cases. Soper and Dunnegan [20, 30] had similar results.

Stones may migrate like sponges or other foreign bodies in the digestive tract [11] as in one of our cases or they can be confused with colonic tumors [2]. Rosin [19] reported stones in a hernia sac. Stones have also been the cause of thoracic empyema [16] and broncopleural fistula with cholelithoptysis [9, 15]. The body tries to expel foreign bodies by the port sites or even trought the bowel or other organs.

Persistent infections or sbucutaneous tumors in the port sites, especially in the umbilicus are generally due to retained stones. Fistulography may show the stones.

Complications due to abandoned stones should be included in grade II type of Strasberg classification [5] because no mortality or lasting incapacity was present.

We agree with Welch [27] and other authors [3, 8, 18] that laparotomy is not routinely indicated when lost stones cannot be retrieved. Only 25% of them will cause complications. However laparotomy should be considered if a large stone or many stones are lost [23]. Laparotomy should be performed if the gallbladder is at large.

Patients should always be informed of stones left in their abdomen, both for legal reasons and for the purpose of early diagnosis of complications.

### CONCLUSIONS

Stones which fall into the abdomen during laparoscopic cholecystectomy may lead to severe complications. Every effort should be made to retrieve them.

Fever, pain, abdominal abscesses or infected sinus tracts at port sites may be result from lost stones, Fistulography, ultrasonography and CT scans should be performed.

As only 25% of those stones produce complications, laparotomy should not be routinely performed to extract them. Patients should be informed in this contingency.

### References

[1] Alcayir, I., Yerdel, M. A., Kubasioglu, U. *et al.* (1995). Clinico-pathological outcome of intraperitonealy retained gall stones with different characteristics. *H. P. B. Surgery*, **9**, Sup. I-5, Athens 95.

[2] Cacdac, R. and Lakra, Y. (1993). Abdominal wall sinus tract secondary to gallstones. A complication of laparoscopic cholecystectomy. *J. Lap. Surg.*, **3**, 509.

[3] Catarci, M., Zaraca, F., Scaccia, M. Y. andCarboni, M. (1993); Lost intraperitoneal stones after laparoscopic cholecystectomy: Harmless sequela or reason for reopertaion? *Surg. Lap. and Endoc.*, **3**, 318.

[4] Corbelle, J. (1992). Discusión del trabajo de Torres, R., Beltrame, O. and y Orban, O. Propuesta de clasificatión laparoscópica de la patología vesicular y su correlación con los resultados quirúrgicos. *Rev. Argent. Cirug.*, **63**, 155, 199.

[5] Clavien, P. A., Sanabria, J. R. and y Strasberg, S. M. (1992). Proposed classification of complications of surgery with examples of utility in cholecystectomy. *Surgery*, **111**, 519.

[6] Diez, J., Delbene, R. and y Ferrers, A. (1995). Análisis de 1000 colecistectomías laparoscópicas. *Rev. Argent. Cirug.*, **68**, 111.

[7] Dittrich, K. and Weis, H. (1995). Ileus of the small intestine caused by a lost gallstone. A late complication of lapraoscopic cholecystectomy. *Chirurg.*, **66**, 443.

[8] Diez, J., Arozamena, C. J., Ferraina, P. A., Franci, J. M., Ferreres, A., Lardies, J. M. and y Gutierrez, V. P. (1996). Relation between postoperative infections and gallbladder bile leakage during laparoscopic cholecystectomies. *Surg. Endosc.*, **10**, 529.

[9] Downie, G., Robbins, M., Souve, J. *et al.* (1993). Cholelithoptysis. A complication of laparoscopic cholecystectomy. *Chest*, **103**, 616.

[10] Guy, P. R., Watkin, D. S. and y Thompson, M. H. (1993). Late discharge of stones after laparoscopic cholecystectomy. *Brit. J. Surg.*, **80**, 1052.

[11] Kaplan, J., Serafini, V., Nespral, E., Taddei, A. M., Zutelman, C. A., Wigutow, N. G. and y Menendez, J. A. (1993). Complicaciones de la colecistectomíca. *Rev. Argent. Cirug.*, **65**, 44.

[12] Kraf, T., Butters, M. and Bittner, R. (1994). The lost gallstone. Complications after laparoscopic cholecystectomy. *Chirurg.*, **65**, 142-3.

[13] Kent, R. B. and y Stahl; R. D. (1991). Laparoscopic Retrieval of spilled stones. *Surg. Lap. and Endosc.*, **2**, 152.

[14] Klaiber, Ch., Metzger, A., Leepin, H. and y Saager, Ch., Die laparoskopische cholecystektomie, 100 konsekutive falle ohne postoperative morbiditat. *Schwiez Med. Wochenschr.*, **121**, 898.

[15] Lee, V., Paulsen, E. and Libby, E. (1993). Cholelithoptisis and cholelithorrea rare complications of laparoscopic cholecystectomy. *Gastroent.*, **105**, 1877.

[16] Leslie, K., Raukin, R. and Duff, J. (1994). Lost stones during laparoscopic cholecystectomy: are they really benign? *Can. J. of Surg.*, **37**, 240.

[17] Paul, A., Eypasch, E. P., Holthausen, U. and Troidl, H. (1995). Bowel obstruction caused by a free intraperitoneal gallstone. A late complication after laparoscopic cholecystectomy. *Surgey*, **117**, 595.

[18] Ponsky, J. L. (1991). Complications of laparoscopic cholecystectomy, *Am. J. Surg.*, **161**, 393.

[19] Rosin, D., Koriausky, Y., Yudichi, A. *et al.* (1995). Lost gallstones found in hernial sac. *Jour. of Laparoend. Surg.*, **50**, 409.

[20] Soper, N. J. and y Dunnegan, D. L. (1991). Does intraoperative gallbladder perforation influence the early outcome of laparoscopic cholecystectomy? *Surg. Lap. and Endosc.*, **1**, 156.

[21] Stewart, L., Smith, A. L., Pellegrini, C. A., Motson, R. W. and y Way, L. W. (1987). Pigment gallstones form as a composite of bacterial microcolonies and pigment solid. *Ann. Surg.*, **206**, 242.

[22] Strasberg, S. and Soper, N. (1996). Complications of laparoscopic general surgery. *Gast. Clin. of N. Amer.*, **6**(2), 423.

[23] Targarona, E. M., Cifuentes, A., Balagué, À. A. *et al.* (1995). The spilled stone, a potential danger after laparoscopic cholecystectomy. *HPB Surgery*, **9**, Sup I, 124 Athens 95.

[24] Trede, M., Mouiel, J. and y Soper, N. (1993). Pitfalls errors in laparoscopic cholecystectomy. State of the art of Surgery 1993/4. Luncheon Panels 35th World Congress of Surgery, *Int. Sur. Soc.*, Hong Kong, 26-07.

[25] van Brunt, P. and Lanzafame, R. (1994). Subhepatic inflamatory mass after laparoscopic cholecystectomy. A delayed complication of spilled stones. *Arch. Surg.*, **129**, 882-3.

[26] Welch, N., Hinder, R., Ciures, T. and y Bacon, N. (1991). Laparoscopic capture of escaped gallstones. *Surg. Lap. and Endosc.*, **1**, 42.

[27] Welch, N., Hinder, R. A., Fitzgibbons, R. J. and y Rouse, J. W. (1991). Gallstones in the peritoneal cavity. A clinical and experimental study. *Surg. Lap. and Endosc.*, **1**, 246.

[28] Wetscher, G., Schnaub, G., Feud, F. *et al.* (1994). Subcutaneous abcess due to gallstones lost during laparoscopic cholecystectomy. *Endosc.*, **26**, 324.

[29] Wills, V. and Smith, R. (1995). Gallstone ileus post cholecystectomy. **64**, 650-652.

[30] Wilton, P., Peters, J., Thomas, C. *et al.* (1993). Laparoscopic cholecystectomy. Leave no spilled stone unturned. *Surg. Endosc.*, **7**, 537-8.

[31] Zucker, K. (1993). Surgical laparoscopy upate - 125 Quality Medical Publishing Inc. St Louis Missouri.

### COMMENTARY

''What experience and history teach is this – that people and governments never have learned anything from history, or acted upon the principles deduced from it' GWF Hegel 1770-1831, Philosophy of History.

Whilst this quotation is a little harsh to describe surgical practice and the influence of our surgical forefathers, the revolution in minimally invasive surgery that has occurred over the past 10 years has in certain areas reflected Hegel's point of view. Following the introduction of laparoscopic cholecystectomy in the late 1980's, a certain complacency crept into some aspects of surgical practice. Many surgeon's attitudes to operative cholangiography changed as did their opinion of the need to recover stones "lost" within the peritoneal cavity. The data presented by Diez and colleagues is the latest in a series of papers showing that lost stones are not innocuous and that every attempt should be made to recover them at the time of laparoscopy. The overall risk of complications arising from lost stones is not high but given the fact that most if not all of these complications are avoidable the message is very clear. We must return to the lessons taught us in the era of open cholecystectomy, namely that every effort must be made to remove stones and bile spilt during cholecystectomy."

Mr. I. G. Martin
Academic Department of Surgery
The General Infirmary at Leeds
Great George Street
LEEDS
LS1 3EX

